# Heterostructure of NiFe@NiCr-LDH for Active and Durable Oxygen Evolution Reactions in Alkaline Media

**DOI:** 10.3390/ma16082968

**Published:** 2023-04-08

**Authors:** Sanchuan Liu, Yujun Tang, Chengyu Guo, Yonggang Liu, Zhenghua Tang

**Affiliations:** 1 New Energy Research Institute, School of Environment and Energy, South China University of Technology, Guangzhou Higher Education Mega Center, Guangzhou 510006, China; 2 State Key Laboratory of Subtropical Building Science, South China University of Technology, Guangzhou 510640, China

**Keywords:** heterostructure, NiFe@NiCr-LDH, oxygen evolution reaction, synergistic effect, long-term stability

## Abstract

Developing cost-effective, efficient, and durable catalysts for oxygen evolution reactions (OER) is the key for promoting large-scale H_2_ production through electrochemical water splitting. Herein, we report a facile method for fabricating an NiFe@NiCr-LDH catalyst toward alkaline OER. The electronic microscopy technique revealed that it has a well-defined heterostructure at the interface between the NiFe and NiCr phases. In 1.0 M KOH, the as-prepared NiFe@NiCr-LDH catalyst shows excellent catalytic performance, evidenced by an overpotential of 266 mV at the current density of 10 mA cm^−2^ and a small Tafel slope of 63 mV dec^−1^; both are comparable with the RuO_2_ benchmark catalyst. It also exhibits robust durability in long-term operation, manifested by a 10% current decay in 20 h, which is superior to that of the RuO_2_ catalyst. Such excellent performance is attributed to the interfacial electron transfer that occurs at the interfaces of the heterostructure, and the Fe(III) species facilitate the formation of Ni(III) species as active sites in NiFe@NiCr-LDH. This study offers a feasible strategy for preparing a transition metal-based LDH catalyst for OER toward H_2_ production and other electrochemical energy technologies.

## 1. Introduction

Promoting green energy technologies with low-carbon or even carbon-free emission is essential to resolve the global warming challenge and ubiquitous environmental issues [1,2]. H_2_ has been widely regarded as one of the most promising green energy sources because it has an ultrahigh energy density and does not produce any carbon emission when using it [3,4]. Electrochemical water splitting has been proven to be one effective strategy for producing high-quality H_2_, yet developing efficient and durable catalysts for two half reactions including hydrogen evolution reaction (HER) and oxygen evolution reaction (OER) is critical [5,6,7,8]. Nevertheless, compared to HER, the sluggish kinetics of OER, which has a more compldata nex four-electron transfer process, are the bottleneck that determines the water electrolysis efficiency [9,10]. Currently, RuO_2_ and IrO_2_ are the benchmark catalysts for OER, but the low abundance in Earth’s crust has significantly restricted their large-scale commercial application [11,12,13,14]. Moreover, both RuO_2_ and IrO_2_ are suffering from other issues that are detrimental to the practical application, e.g., RuO_2_ can form RuO_4_^2−^ and becomes dissolved under a high positive potential in the OER process, thus resulting in poor durability [15,16]. To that end, developing low-cost, efficient, and durable OER catalysts is imperative for realizing practical water splitting toward H_2_ generation.

Recently, transition metal layered double hydroxides (LDHs, whose chemical formula is denoted as [M(II)_1−x_M(III)_x_(OH)_2_][A^n−^]_x/n_·yH_2_O, where M(II) can be Ni^2+^, Co^2+^, Zn^2+^, etc., M(III) can be Fe^3+^, Cr^3+^, V^3+^, etc., and A^n−^ is the charge balancing anion) have been attracting considerable interest as promising OER catalysts, thanks to their relatively low cost, their facile synthesis, and, more importantly, the well-defined two-dimensional structure that induces abundant active sites that can be exposed for OER [17,18,19,20]. Among these, NiFe-LDHs are the most widely explored, not only because of the high abundance of the Ni/Fe elements but also due to the fact that numerous NiOOH active species can be generated at the edge of NiFe-LDHs during the OER process [21,22,23]. To enhance the OER performance of NiFe-LDHs, various strategies have been documented, including morphology control, doping, defect engineering, intercalation, exfoliation, and so on [24,25,26,27,28,29]. A previous study revealed that introducing a third metal can lead to the charge redistribution and hence may enhance the OER performance [30,31,32]. For instance, Zhang et al. reported a catalyst of single Au atoms supported on NiFe-LDH (denoted as ^s^Au/NiFe LDH), which had a small overpotential of 0.21 V at the current density of 10 mA cm^−2^ in 1 M KOH for OER; DFT calculation demonstrated that the Au single atoms can induce the formation of an active NiFeOOH layer on the LDHs’ edges, leading to improved adsorption energies of the OER intermediates on the Fe active sites [33]. In another study, Feng’s group prepared a ruthenium catalyst anchored on the surface of CoFe-LDHs (denoted as Ru/CoFe-LDHs), and it showed an overpotential of 198 mV at 10 mA cm^−2^ and a small Tafel slope of 39 mV dec^−1^ in 1.0 M KOH for OER; Operando X-ray absorption spectroscopy (XAS) disclosed that Ru maintained the oxidation state of IV in the OER process due to the optimal synergistic electron coupling, and such highly valent Ru species served as the active center [34].

Despite the above progress, the OER performance of NiFe-LDHs is still restricted by its physiochemical properties—particularly, the insulation properties of nickel hydroxides limit the charge transfer capability [22,27,35]. Therefore, the appropriate adjustment of the electronic structure of nickel hydroxides to improve the electric conductivity holds great potential for enhancing the electrocatalytic performance [36,37,38]. Cr(III) cations occupying the octahedral center (NiFe-LDHs have numerous such centers) present a unique electronic configuration (t_2g_^3^e_g_^0^) which can accelerate the charge transfer and hence can be beneficial for the OER process [38,39,40,41,42,43]. For example, Jin’s team synthesized a stable trimetallic NiFeCr-LDHs (molar ratio Ni:Fe:Cr = 6:2:1) catalyst on conductive carbon paper, and it demonstrated superior OER performance to that of NiFe-LDHs, with a low overpotential of 225 mV at a current density of 25 mA cm^−2^, thanks to the synergistic interaction in the metal center [44]. In another study, the Chen group developed NiCr-LDH as a bifunctional electrocatalyst for overall water splitting, and the optimized sample of Ni_2_Cr_1_-LDH showed extraordinary OER activity, evidenced by an ultralow overpotential of 319 mV at 100 mA cm^−2^ and outstanding durability at 1.55 V (vs. RHE) for 30 h in 1 M KOH; DFT calculations disclosed that the Cr^3+^ ions within the LDH layer served as charge transfer sites and thus boosted the intrinsic electrochemical activity [45].

Inspired by the above findings, herein, we report Ni-Fe and Ni-Cr coupled LDHs with a heterostructure (NiFe@NiCr-LDH) through a facile two-step hydrothermal approach as a high-efficiency OER catalyst. A series of characterizations revealed that a well-defined heterostructure is formed between the NiFe-LDH and NiCr-LDH phases in NiFe@NiCr-LDH. NiFe@NiCr-LDH exhibited excellent OER performance in 1 M KOH, manifested by a low overpotential of 266 mV at 10 mA cm^−2^ and a small Tafel slope of 63 mV dec^−1^, which are both comparable with those of the RuO_2_ benchmark catalyst, as well as superior long-term stability compared to that of RuO_2_. The XPS analysis before and after the OER test disclosed that the outer shell Fe(III) species facilitate the oxidation of Ni(II) into Ni(III) as active sites for OER, which cannot be achieved with NiCr@NiFe-LDH and other control samples.

## 2. Experimental Section

### 2.1. Materials

Ruthenium dioxide (RuO_2_) and 5% Nafion perfluorinated resin were bought from the Aladdin chemical company. Nickel (II) nitrate hexahydrate (Ni(NO_3_)_2_·6H_2_O, 99%), iron (III) nitrate nonahydrate (Fe(NO_3_)_3_·9H_2_O, 99%), chromium (III) nitrate nonahydrate (Cr(NO_3_)_3_·9H_2_O, 99%), sodium fluoride (NaF, ≥98.0%), and ethanol (99.0%) were purchased from Energy Chemical Company, China. Urea (CO(NH_2_)_2_, ≥99%) and ammonium fluoride (NH_4_F, >99%) were purchased from Macklin Chemical Company. All solutions were prepared using deionized water (DIW, the resistivity is 18.2 MΩ cm^−1^ at room temperature). All the chemicals were used as received, without further purification.

### 2.2. Synthesis of NiCr-LDH

NiCr-LDH was synthesized by following a modified hydrothermal approach [30,45,46]. Typically, 0.8 mL of Ni(NO_3_)_2_ solution (1.0 M) and 0.8 mL of Cr(NO_3_)_3_ solution (0.5 M) were mixed together into 3.4 mL DIW to form Solution A. Meanwhile, 5 mmol urea, 1 mmol NaF, and 2 mmol NH_4_F were added into 10 mL DIW under mild stirring for 5 min to prepare Solution B. Solutions A and B were then mixed and transferred into a 25 mL Teflon autoclave. Then, the liner was heated at 120 °C for 6 h, during which NiCr-LDH nanospheres slowly precipitated out. After that, the mixture was centrifuged and washed by DIW and ethanol, respectively, several times. After being dried overnight in vacuum, wathet blue powder as the final product was collected and denoted as NiCr-LDH.

### 2.3. Synthesis of NiFe@NiCr-LDH

**Scheme 1:**
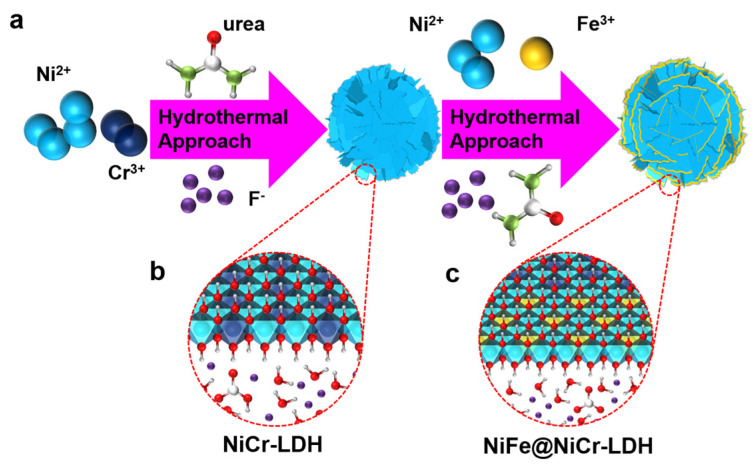
(**a**) Schematic illustration for synthesizing NiFe@NiCr-LDH, where cyan, indigo-blue, yellow, purple, red, green, white, and gray atoms represent Ni, Cr, Fe, F, O, N, C, and H, respectively; Ideal models for (**b**) NiCr-LDH and (**c**) NiFe@NiCr-LDH with interlayer anions and H_2_O molecules.

On the basis of forming NiCr-LDH, NiFe@NiCr-LDH was further synthesized by a second hydrothermal step similar to the first one (See Scheme 1). Such sequential hydrothermal step is important for constructing a heterostructure between NiFe-LDH and NiCr-LDH instead of forming homogeneous layered triple hydroxide (LTH). Typically, 0.3 mL Ni(NO_3_)_2_ solution (1.0 M) and 0.2 mL Fe(NO_3_)_3_ solution (0.5 M) were added into 4.5 mL DIW to form Solution C. Meanwhile, 5 mmol urea and 3 mmol NH_4_F were added into another 10 mL of DIW under mild stirring for 5 min to prepare Solution D. Solutions C and D were then mixed and transferred into a 25 mL Teflon autoclave. After that, 60 mg NiCr-LDH powder was added into the autoclave with vigorous ultrasonic treatment for 5 min. Subsequently, the autoclave was heated at 120 °C for 2 h, during which NiFe-LDH nanosheets were precipitated on the surface of NiCr-LDH nanospheres. After that, the mixture was centrifuged, washed, and dried. The final product was collected and also denoted as NiFe@NiCr-LDH.

### 2.4. Characterizations

The morphologies of the samples were observed by a field-emission scanning electronic microscope (SEM, Tescan MIRA LMS), a field-emission transmission electronic microscope (TEM, Talos F200X operating at 200 kV), and high-angle annular dark-field scanning TEM (HAADF-STEM operating at 200 kV), and the elemental mapping was analyzed by energy dispersive spectroscopy (EDS, Tencai G2 F20 X-Twin). The mass content of the Ni, Cr, Fe, and O elements was determined by inductive coupled plasma optical emission spectroscopy (ICP-OES, Agilent 720ES). The physiochemical properties of the samples were characterized by high-resolution TEM (HRTEM), selected area electron diffraction (SAED, Talos F200X), Fourier transform infrared spectroscopy (FTIR, Thermo Nicolet iS 10), X-Ray Diffraction (XRD, Rigaku SmartLab using Cu K_α_ radiation at 10° min^−1^ in the 2θ range of 5°–90°), and X-ray photoelectron spectroscopy (XPS, PHI 5000 Versaprobe III).

### 2.5. Electrochemical Tests

All the electrochemical measurements were conducted on a CHI760D electrochemical workstation (Chenhua Instruments Co. Ltd., China) with a three-electrode standard system in 1.0 M KOH aqueous solution. The catalysts cast on a glassy carbon electrode (GCE) was adopted as the working electrode, which was prepared as follows: 5 mg of each sample was first dispersed in 1 mL solution made of 0.04 mL of Nafion, 0.24 mL of water, and 0.72 mL of ethanol. Then, the mixture was sonicated for 30 min to obtain the uniform suspension. Subsequently, 5 μL of the dispersion was dropwise cast onto GCE and dried to make a working electrode. A carbon rod and an Hg/HgO electrode were used as the counter electrode and the reference electrode, respectively. All the potentials were calibrated to a reversible hydrogen electrode (RHE) according to the following equation:

E_RHE_ = E_Hg/HgO_ + (0.098 + 0.05916 × pH) V (1)

where E is the potential versus RHE or Hg/HgO electrode.

Linear sweep voltammetry (LSV) polarization curves were recorded at a scan rate of 5 mV s^−1^ with iR compensation. The Tafel slope was calculated based on the following equation:

η = b log j + a(2)

where η is the overpotential, b is the Tafel slope, a is the Tafel intercept, and j is the current density (mA cm^−2^). Electrochemical impedance spectroscopy (EIS) measurements were performed at an overpotential of 50 mV from 100 MHz to 1 Hz with an alternating current amplitude of 5 mV. The electrochemical double-layer capacitance (C_dl_) was determined from the CV curves and measured in a potential range without Faradaic current by C_dl_ = Δj/Δv, where v is the scan rate (20~100 mV s^−1^) and Δj equals half of the difference between the anode and cathode current density (Δj = (j_a_ − j_c_)/2). The long-term durability of the catalyst was investigated by chronoamperometry.

## 3. Results and Discussion

### 3.1. Characterization of the Samples

The NiFe@NiCr-LDH catalyst was first prepared by following the synthetic route in Scheme 1. Briefly, Cr(NO_3_)_3_, Ni(NO_3_)_2_, urea, and NH_4_F were reacted together through a hydrothermal approach to form NiCr-LDH first; then, NiFe-LDH was in situ formed onto it to yield NiFe@NiCr-LDH. In addition, a series of control samples including NiCr-LDH, NiFe-LDH, NiCrFe-LTH, and NiCr@NiFe-LDH were also prepared (Scheme S1). The detailed procedure can be found in the Supplementary Information. The mass loadings of Ni, Cr, and Fe were then determined by ICP-OES to be 35.1 wt.%, 11.0 wt.%, and 4.4 wt.%, respectively (Table S1).

**Figure 1:**
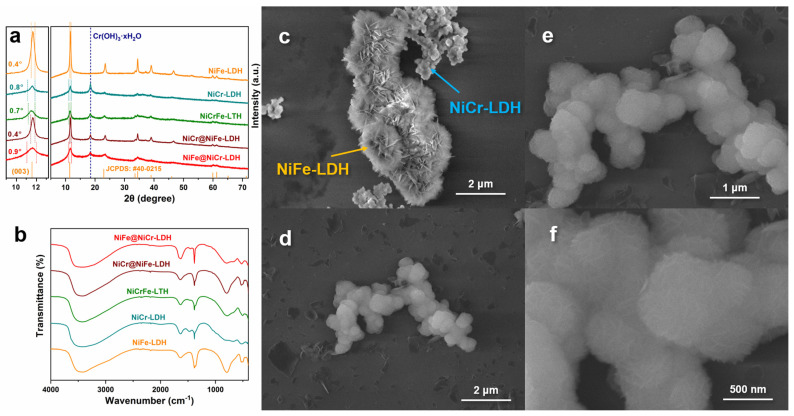
(**a**) XRD patterns of NiFe@NiCr-LDH, NiCr@NiFe-LDH, NiCrFe-LTH, NiCr-LDH, and NiFe-LDH, with FWHM values of LDHs’ (003) peaks; (**b**) FTIR spectra of the as-prepared LDH samples; (**c**) Typical SEM images of NiFe-LDH/NiCr-LDH; (**d**–**f**) Typical SEM images of NiFe@NiCr-LDH with different magnifications.

The XRD patterns of the samples were then probed to obtain the crystal structure information, where the Ni^2+^, Fe^3+^, and/or Cr^3+^ ions occupy the octahedral center of the unit in the LDHs (Scheme 1b-c, Scheme S1c–e). As shown in Figure 1a, all the samples possess the peaks at 11.4°, 23.0°, 33.5°, 34.4°, 39.0°, 59.9°, and 61.2°, which can be attributed to the (003), (006), (101), (012), (015), (110), and (113) crystal planes of the LDHs (JCPDS no. 40-0215), respectively [30]. Moreover, the intensity of the peaks in NiFe-LDH is much higher than that of NiCr-LDH, while the FWHM values of the peaks in NiFe-LDH are lower than those of the other samples, even though the different LDH phases have a similar structure. It suggests that, the introduction of Cr(III) can significantly reduce the LDH’s crystallinity [47]. In addition, the peak of hydrated chromium hydroxide (Cr(OH)_3_·xH_2_O) located at 18.5° was detected in all the Cr(III)-containing LDH samples [48]. The hydroxide may partly dissolve in base media and form pores in LDHs, which is probably beneficial for exposing active sites for OER. FTIR spectroscopy was then performed to detect the interlayer ions and molecules. As shown in Figure 1b, the broad strong absorption band from 3700 cm^−1^ to 2500 cm^−1^ can be attributed to the stretching vibration of the O-H group. The peak at ~1651 cm^−1^ is assigned to the bending vibration of interlayer bound water, indicating the existence of hydrogen bonds. Other interlayer anions may include CO_3_^2−^ or HCO_3_^−,^ and the corresponding sharp stretching vibration absorption peaks are located at ~1384 cm^−1^. The wide peak located at ~794 cm^−1^ demonstrates the existence of F^−^ [44]. Therefore, CO_3_^2−^, HCO_3_^−^, and F^-^ are the main interlayer anions of all the LDH samples. The absorption peaks below 800 cm^−1^ are related to the bending and stretching modes of M-OH and M-OM in the brucite-like layers (M = Ni, Cr, or Fe); yet, the peak intensity can distinguish the NiFe-LDH phase and NiCr-LDH phase in the samples [42]. Nevertheless, NiCr@NiFe-LDH mainly inherits the feature from NiFe-LDH, while NiFe@NiCr-LDH preserves the characteristics from NiCr@LDH.

The morphology of the samples was then examined by the SEM technique. From low-magnification SEM images of NiFe-LDH and NiCr-LDH (Figure 1c and Figure S1), it can be noted that both NiFe-LDH and NiCr-LDH show a nanosheet primary structure, spherical secondary structure, and coral-like tertiary structure [49]. One distinction between them is that NiFe-LDH consists of vivid nanosheets, while NiCr-LDH has vague ones because of its lower crystallinity [45]. Another difference is the size of these nanospheres: the NiFe-LDH nanospheres’ diameter is ~2 μm, but the NiCr-LDH nanospheres’ diameter is smaller than 500 nm. Further, the other three samples have a distinctive morphology and vivid phase structure. The SEM images of the NiFe@NiCr-LDH nanospheres (Figure 1d–f) exhibit a combined morphology feature: relatively small NiFe-LDH nanosheets grown on the surface of NiCr-LDH spheres (1 μm); hence, NiFe@NiCr-LDH adopts the structural feature of both NiFe-LDH and NiCr-LDH. In comparison, NiCr@NiFe-LDH (Figure S2a–c) has a vague primary structure covered by external NiCr-LDH phases (Figure S1d–f) compared to that of NiFe-LDH (Figure S1a–c); NiCrFe-LTH (Figure S2d–f) has an NiFe-LDH-like morphology with a homogeneous structure that is quite different from heterogeneous NiFe@NiCr-LDH or NiCr@NiFe-LDH [50].

**Figure 2:**
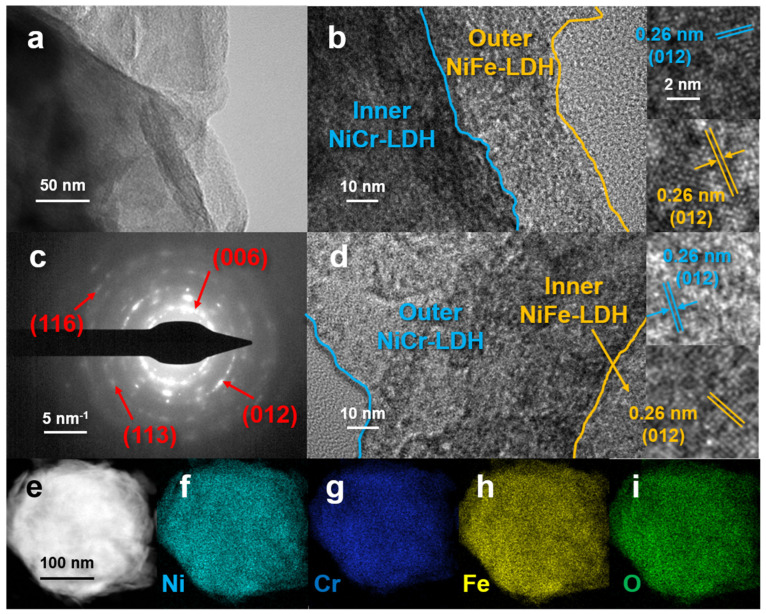
(**a**) Typical TEM image, (**b**) HR-TEM images along with lattice fringes of NiCr-LDH and NiFe-LDH phases in NiFe@NiCr-LDH, (**c**) SAED pattern of NiFe@NiCr-LDH nanosheets, (**d**) Typical HR-TEM images of NiCr@NiFe-LDH nanosheets along with lattice fringes of NiCr-LDH and NiFe-LDH phases, (**e**) HAADF-STEM image of an NiFe@NiCr-LDH nanosphere, (**f**–**i**) Elemental mapping of Ni, Cr, Fe, and O elements of the NiFe@NiCr-LDH nanosphere.

The detailed microstructure of the samples was then scrutinized by TEM and high-resolution TEM (HR-TEM) techniques. Figure 2a shows that the NiFe@NiCr-LDH nanosheets are stacked together, while similar stacking phenomena can also be observed in NiCr@NiFe-LDH and NiCrFe-LTH (Figure S3a–c) [30]. Further, the HR-TEM analysis can clearly differentiate the different phases (Figure S4e), as they possess different crystallinity [22]. As illustrated in Figure 2b,d, the NiCr-LDH phase (with vague lattice fringes in all places) and the NiFe-LDH phase (with well-defined lattice fringes everywhere) have identifiable boundaries, suggesting that a heterostructure is formed in both NiFe@NiCr-LDH and NiCr@NiFe-LDH. Interestingly, the lattice spacing of the NiFe-LDH (012) crystal phase is identical with that of the NiCr-LDH (012) phase. In contrast, NiCrFe-LTH displays homogeneous crystallinity (Figure S3d), reflecting its homogeneous structure [47]. In addition, the SAED of NiFe@NiCr-LDH (Figure 2c) exhibits the combined features, where the diffraction rings are from the crystal planes of small-sized NiCr-LDH phases and the diffraction spots are mainly attributed to the large and integral NiFe-LDH’s crystal phases, as observed in the SEM images and an HR-TEM image (Figure S4e) [42]. Therefore, NiFe@NiCr-LDH consists of primary NiCr-LDH phases and secondary NiFe-LDH phases. Instead, NiFe-LDH phases are dominant in NiCr@NiFe-LDH (Figure S4a), and the presence of more vague diffraction rings in NiCrFe-LTH’s pattern (Figure S4b) is due to the fact that Cr(III) is ubiquitous in the whole sample. Furthermore, such structural pattern in NiFe@NiCr-LDH is verified by the HAADF-STEM image and elemental mapping analysis (Figure 2e–i) [48]. One may notice that the Fe element has a somehow wider distribution region than the Cr element, indicating that NiFe@NiCr-LDH has more external Cr and internal Fe elements. In contrast, although the mass loading of Fe is higher than that of Cr in NiCr@NiFe-LDH, more Cr elements are observed in this sample, suggesting that it is in the outer layer (Figure S5), while the Ni, Cr, and Fe elements are homogeneously distributed in the NiCrFe-LTH sample (Figure S6). More importantly, the EDS line-scanning profile covers a whole NiFe@NiCr-LDH nanosphere (Figure S4c,d), and the elemental distribution can be clearly identified. As depicted in Figure S4d, both Ni and Cr have a peak shape distribution, but the intensity of Ni is much higher than that of Cr, suggesting that Ni is everywhere in the whole sample but Cr is mainly located in the center, and, more importantly, Fe has a slightly higher intensity at the edges than in the middle, suggesting it has more content in the outer layer.

**Figure 3:**
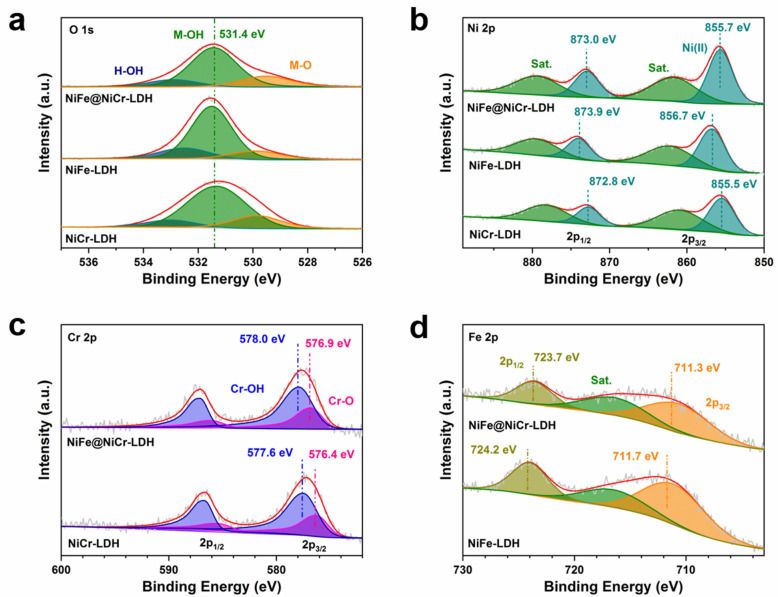
High-resolution XPS spectra of the (**a**) O 1s, (**b**) Ni 2p, (**c**) Cr 2p, and (**d**) Fe 2p electrons in NiFe@NiCr-LDH, NiCr-LDH, and NiFe-LDH.

Subsequently, the chemical composition and valence state of the samples were investigated by X-ray photoelectron spectroscopy (XPS). The existence of the Ni, Cr, Fe, C, O, and F elements can be proved distinctly in survey-scan spectra (Figure S7a). Further, more detailed structural information can be obtained through the high-resolution XPS spectra. In the core-level O 1s spectra (Figure 3a), the metal hydroxide (M-OH) species is identified at the peak binding energy of 531.4 eV, and interlayer bound water (H-OH) and metal oxide (M-O) species are also observed [51]. Nevertheless, the binding energy of the O 1s electrons exhibited no significant difference in NiFe-LDH, NiCr-LDH, and NiFe@NiCr-LDH, indicating that the M-OH species is dominant in the three samples. In the high-resolution C 1s (Figure S7b) and F 1s (Figure S7c) spectra of each sample, the presence of O-C=O (288.9 eV) and F^−^ (684.8 eV) can be readily identified in good echo with the findings in the XRD measurement, further attesting to the presence of these interlayer anions [44].

The high-resolution Ni^2+^ 2p spectra of the NiCr-LDH, NiFe-LDH, and NiFe@NiCr-LDH are presented in Figure 3b [52]. The binding energies of the Ni^2+^ 2p_3/2_ and 2p_1/2_ electrons in NiFe@NiCr-LDH are 855.7 eV and 873.0 eV, respectively, which are slightly higher than that in NiCr-LDH and lower than that in NiFe-LDH. It suggests that there is more electron transfer from Ni(II) to Fe(III) than from Ni(II) to Cr(III) because of iron’s higher electronegativity compared to chromium, and this results in the facile tendency of marginal exposed Ni(II) species being oxidized into the OER active Ni(III) species. Such charge transfer behaviors are further evidenced by the high-resolution Cr^2+^ and Fe^2+^ spectra [53]. As illustrated in Figure 3c, the deconvoluted Cr^2+^ 3p_3/2_ peak shows a doublet, in which the binding energy at 578.0 eV and 576.9 eV is ascribed to chromium hydroxide (Cr-OH) and chromium oxide (Cr-O), respectively. Both values are slightly higher than that from NiCr-LDH, implying that Cr (III) gains fewer electrons from Ni (II) in NiFe@NiCr-LDH. Similarly, the binding energy of the Fe 2p_3/2_ electrons (Figure 3d) decreased by ~0.4 eV for NiFe@NiCr-LDH (711.3 eV) as compared to that of NiFe-LDH (711.7 eV). Previous studies have revealed that such rational electron transfer from the low valent metals to the high valent metals can cause charge distribution in the catalyst and is hence favorable for facilitating the electrocatalytic kinetics [49].

### 3.2. Electrochemical Performance toward OER

**Figure 4:**
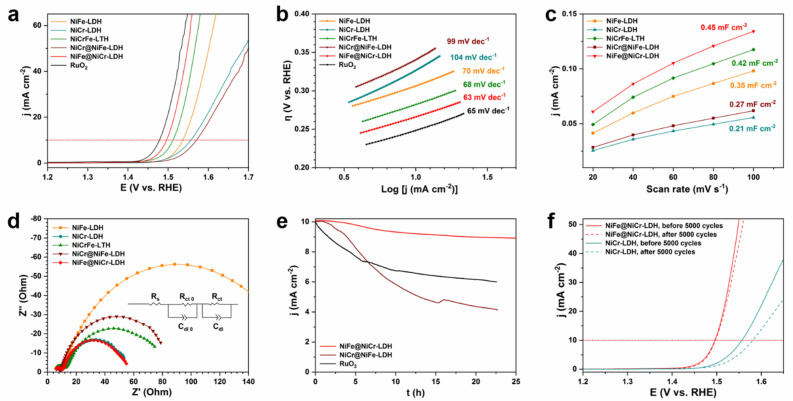
(**a**) LSV polarization curves and the corresponding Tafel plots (**b**) of NiFe@NiCr-LDH, NiCr@NiFe-LDH, NiCrFe-LTH, NiCr-LDH, NiFe-LDH, and RuO_2_ in 1.0 M KOH; (**c**) The current density at 1.18 V vs. the scan rate for NiFe@NiCr-LDH, NiCr@NiFe-LDH, NiCrFe-LTH, NiCr-LDH, and NiFe-LDH; (**d**) Electrochemical impedance spectra (EIS) of the samples; (**e**) The i-t curve at the current density of 10 mA cm^−2^ for 24 h; (**f**) The LSV polarization curves of the NiFe@NiCr-LDH and NiCr-LDH catalyst before and after 5000 CV cycles.

The electrochemical performance of the catalysts toward oxygen evolution reaction (OER) was performed next in O_2_-saturated 1.0 M KOH aqueous solution [54]. As presented in Figure 4a, the polarization curves of the series of samples distinguish their OER activities. To afford a current density of 10 mA cm^−2^, the required overpotential is 266 mV for NiFe@NiCr-LDH, which is close to that of the benchmark RuO_2_ (249 mV) catalyst. This value is also much lower than that of NiCr-LDH (327 mV) and NiCr@NiFe-LDH (340 mV), indicating more active sites on the external NiFe-LDH phase instead of the inner NiCr-LDH phase. The overpotential value of NiFe@NiCr-LDH is also smaller than that of NiCrFe-LTH (282 mV) and NiFe-LDH (306 mV), indicating that the introduction of Cr(III) and its location are both crucial for enhancing the OER performance. The reaction kinetics were then analyzed by Tafel plots, where the Tafel slope values were extrapolated (Figure 4b). NiFe@NiCr-LDH has a small Tafel slope of 63 mV dec^−1^, which is lower than that of NiCrFe-LTH (68 mV dec^−1^) and NiFe-LDH (70 mV dec^−1^), indicating rapid OER kinetics. The Tafel slope values of NiCr-LDH (104 mV dec^−1^) and NiCr@NiFe-LDH (99 mV dec^−1^) are higher because of the limitation from the external NiCr-LDH phase. Furthermore, the OER activity of NiFe@NiCr-LDH outperforms or is at least comparable to recently reported LDH catalysts with a similar brucite-like layered structure under the same reaction conditions, as summarized in Table S2 [38,44,47,48,49,50,51,52,53,54,55].

To probe the underlying intrinsic reason for the brilliant OER performance of NiFe@NiCr-LDH, the electrochemically active surface area (ECSA) of all the samples was then evaluated (Figures S8 and S9) [34]. It is known that the ESCA value is linearly related to the double layer capacitance (C_dl_) in the non-Faradaic potential region. As presented in Figure 4c, NiFe@NiCr-LDH has a C_dl_ value of 0.45 mF cm^−2^, which is larger than that of the other samples, indicating more exposed active sites for electrocatalysis. Moreover, the reaction kinetics was also examined by electron impedance spectroscopy (EIS), where the charge transfer behaviors during the OER process can be appraised. As illustrated in Figure 4d, NiFe@NiCr-LDH has a quite small semi-cycle in the series, suggesting rather fast reaction kinetics. By using an equivalent circuit, the EIS spectra are fitted and shown in the inset of Figure 4d, and all the calculated values are compiled in Table S3. NiFe@NiCr-LDH has the smallest charge transfer resistivity, indicating the most favorable charge transfer capability. In addition, the corresponding fitting C_dl_ value of NiFe@NiCr-LDH (0.44 mF cm^−2^) is also the highest among all the samples, and such high capacitance can facilitate the OER process as well.

Long-term durability is another critical parameter for assessing the intrinsic electrocatalytic performance of the catalyst for prolonged operation [55]. As depicted in Figure 4e, after about 20 h of continuous chronoamperometric testing, the initial current density of NiFe@NiCr-LDH only decreased by about ~11%, while, in stark contrast, NiCr@NiFe-LDH dropped by over 50%, and RuO_2_ also decreased by ~40%. That main location of the metal elements also affects the samples’ OER durability. The markedly outperformed long-term stability of NiFe@NiCr-LDH was further validated by the accelerated durability test (ADT). As shown in Figure 4f, after 5000 cycles of potential scanning, a negligible potential shift at the current density of 10 mA cm^−2^ was observed for NiFe@NiCr-LDH, while NiCr-LDH exhibited a positive overpotential shift of 24 mV. In addition, the EIS spectra before and after the OER test can further verify the outstanding stability of NiFe@NiCr-LDH [17]. As presented in Figure S10 and Table S3, the charge transfer kinetics remained almost unchanged after the OER test in NiFe@NiCr-LDH, but for the other samples, much more sluggish charge transfer kinetics were observed. Moreover, the corresponding fitting C_dl_ value of NiFe@NiCr-LDH exhibited a negligible change (0.43 mF cm^−2^), but for the other samples, the C_dl_ values decreased substantially. In other words, the exposed outer NiCr-LDH phases would reduce the samples’ OER long-term durability.

**Figure 5:**
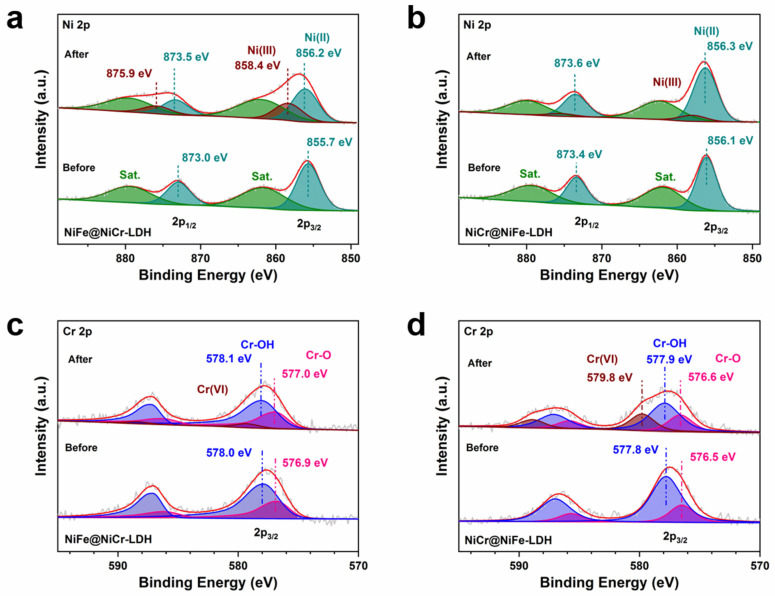
High-resolution XPS spectra of the Ni 2p electrons in (**a**) NiFe@NiCr-LDH and (**b**) NiCr@NiFe-LDH before and after the OER test; High-resolution XPS spectra of the Cr 2p electrons in (**c**) NiFe@NiCr-LDH and (**d**) NiCr@NiFe-LDH before and after the OER test.

To further probe the physical origin of the outstanding activity and stability of the NiFe@NiCr-LDH catalyst, XPS analysis was conducted before and after the OER test [43]. As depicted in Figure 5a, the binding energy of both the Ni^2+^ 2p_1/2_ and Ni^2+^ 2p_3/2_ electrons in NiFe@NiCr-LDH shifted positively after the OER test, and more importantly, Ni^3+^ species also appeared, while in sharp contrast, for NiCr@NiFe-LDH (Figure 5b), the binding energy of both the Ni^2+^ 2p_1/2_ and Ni^2+^ 2p_3/2_ electrons exhibited a negligible change without the presence of Ni^3+^ species. This indicates that, in the presence of Fe^3+^, the Ni^2+^ species becomes partially oxidized in the outer shell in NiFe@NiCr-LDH; however, the Cr^3+^ species is not able to promote the oxidation of Ni^2+^ in the outer shell of NiCr@NiFe-LDH. Meanwhile, despite the binding energy of the Cr(-O) and Cr(-OH) 2p electrons remaining almost unchanged after the OER process in both NiFe@NiCr-LDH (Figure 5c) and NiCr@NiFe-LDH (Figure 5d), the Cr^6+^ species is somehow present in NiCr@NiFe-LDH but cannot be found in NiFe@NiCr-LDH [51]. In other words, Cr^3+^, instead of Ni^2+^ species, becomes partially oxidized in the outer shell in NiCr@NiFe-LDH. In addition, as described in Figure S11, the binding energy of both the Fe^3+^ 2p_3/2_ and Fe 2p_1/2_ electrons increased over 1 eV in NiFe@NiCr-LDH, while the shift in NiCr@NiFe-LDH is negligible, suggesting that Fe^3+^ species in different locations (inner or outer shell) play markedly different roles [50]. When the NiCr phase is exposed (NiCr@NiFe-LDH), Cr(III) becomes easily oxidized into Cr(VI), but Ni still maintains +2 valence; hence, the OER activity cannot be promoted, while, in contrast, the Fe(III) species in the outer shell (NiFe@NiCr-LDH) facilitate the oxidation of Ni(II) into active Ni(III), and Cr(III) is still well-preserved during the OER process to maintain the good conductivity. Such synergistic effect (outer NiFe-LDH phases provide activity; inner NiCr-LDH phases guarantee stability) boosts the NiFe@NiCr-LDH’s OER performance.

## 4. Conclusions

In summary, we developed an NiFe@NiCr-LDH catalyst with heterogeneous interfaces for alkaline OER. Multiple characterizations revealed that a well-defined heterostructure is formed at the interface between the outer NiFe-LDH phase and the inner NiCr-LDH phase. In 1 M KOH, NiFe@NiCr-LDH showed excellent OER performance, evidenced by a low overpotential at 10 mA cm^−2^ and a small Tafel slope, both close to those of the benchmark RuO_2_ catalyst, as well as markedly superior long-term stability compared to that of RuO_2_. Such impressive performance is attributed to the fact that the Fe(III) species in the outer shell accelerate the formation of Ni(III) species as active sites for OER in NiFe@NiCr-LDH, which cannot be achieved for NiCr@NiFe-LDH and other samples without a heterostructure. This study provides a facile strategy for constructing an NiFe-LDH-based heterostructure for OER and other electrochemical energy technologies.

## Data Availability

The authors will supply the relevant data in response to reasonable requests.

## Supplementary Materials

The following supporting information can be downloaded at: https://www.mdpi.com/article/10.3390/ma16082968/s1.
