# Supplementary material for: Heterostructure of NiFe@NiCr-LDH for Active and Durable Oxygen Evolution Reactions in Alkaline Media
Source: Materials (Basel). 2023 Apr 8;16(8):2968. doi: 10.3390/ma16082968 (PMC10142980; doi:10.3390/ma16082968)
Supplement: Supplementary file 1 [file materials-16-02968-s001.zip › materials-2300810-supplementary.pdf]

Supplementary Information

Heterostructure of NiFe@NiCr-LDH for Active and Durable Oxygen Evolution Reactions in Alkaline Media

Sanchuan Liu [1], Yujun Tang [1], Chengyu Guo [1], Yonggang Liu [1] and Zhenghua Tang [1,2,*]

1 New Energy Research Institute, School of Environment and Energy, South China University of Technology, Guangzhou Higher Education Mega Centre, Guangzhou 510006, China
2 State Key Laboratory of Subtropical Building Science, South China University of Technology, Guangzhou 510640, China
* Correspondence: zhht@scut.edu.cn

Details of the preparation of the samples

1 Synthesis of NiFe-LDH

NiFe-LDH was synthesized by following a modified hydrothermal approach. Typically, 0.9 mL $Ni(NO_3)_2$ solution (1.0 M) and 0.6 mL $Fe(NO_3)_3$ solution (0.5 M) were added into 3.5 mL DIW to form Solution A [1]. Meanwhile, 5 mmol urea and 3 mmol NH_4F was added into 10 mL DIW under mild stirring for 5 min to prepare Solution B. Solution A and B was then mixed and transferred into 50 mL Teflon autoclave. Subsequently, the liner was heated at 120 °C for 6 h, during which NiFe-LDH were slowly precipitated in the weak acid media. After that, the mixture was centrifuged, and washed by DIW and ethanol, respectively, for several times. After dried overnight in vacuum, light yellow powder as the final product was collected and denoted as NiFe-LDH.

2 Synthesis of NiCrFe-LTH

The preparation of NiCrFe-LTH followed the similar manner with that of NiFe-LDH. The only difference is that the Solution A was prepared by mixing 0.9 mL $Ni(NO_3)_2$ solution (1.0 M), 0.3 mL $Cr(NO_3)_3$ solution (0.5 M), 0.6 mL $Fe(NO_3)_3$ solution (0.5 M), with 3.5 mL DIW.

3 Synthesis of NiCr@NiFe-LDH

The preparation of NiCr@NiFe-LDH also followed the similar manner with that of NiFe@NiCr-LDH. NiFe-LDH was used as the precursor for preparing NiCr@NiFe-LDH. Solution C was prepared by mixing 0.3 mL $Ni(NO_3)_2$ solution (1.0 M), 0.3 mL $Cr(NO_3)_3$ solution (0.5 M), with 4.4 mL DIW. Solution D was the mixture of 5 mmol urea, 1 mmol NaF, 2 mmol NH_4F, and 10 mL DIW. Prior to the second hydrothermal step, 60 mg NiFe-LDH powder was added in the mixture of Solution C and Solution D under vigorous ultrasonic treatment for 5 min. The final product was collected and denoted as NiCr@NiFe-LDH.

Supplementary Figures

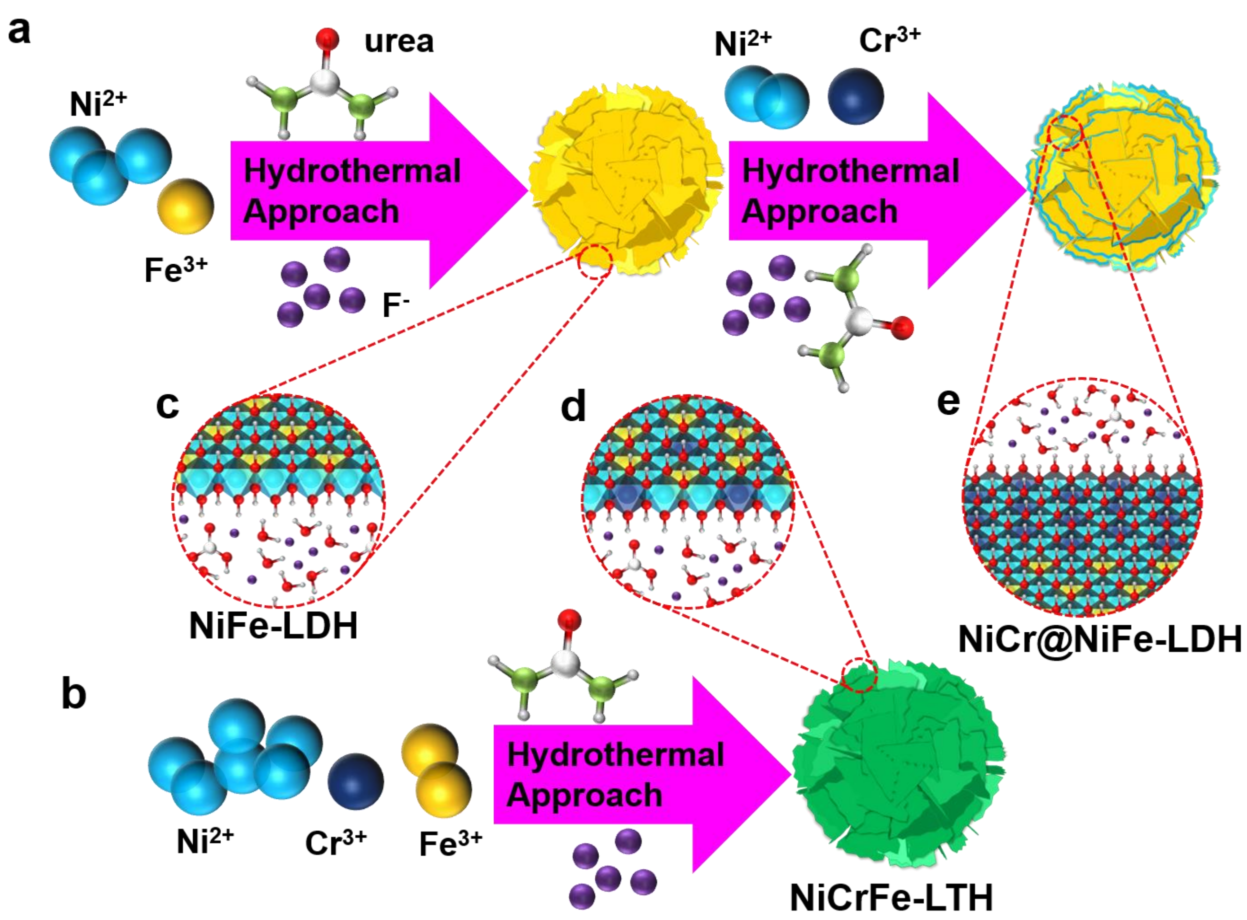

Scheme S1. Schematic illustration for synthesizing (**a**) NiCr@NiFe-LDH and (**b**) NiCrFe-LTH, where cyan, indigo-blue, yellow, purple, red, green, white, and gray atoms represent Ni, Cr, Fe, F, O, N, C, and H, respectively; Ideal models for (**c**) NiFe-LDH, (**d**) NiCrFe-LTH, (**e**) NiCr@NiFe-LDH with interlayer anions and H_2O molecules.

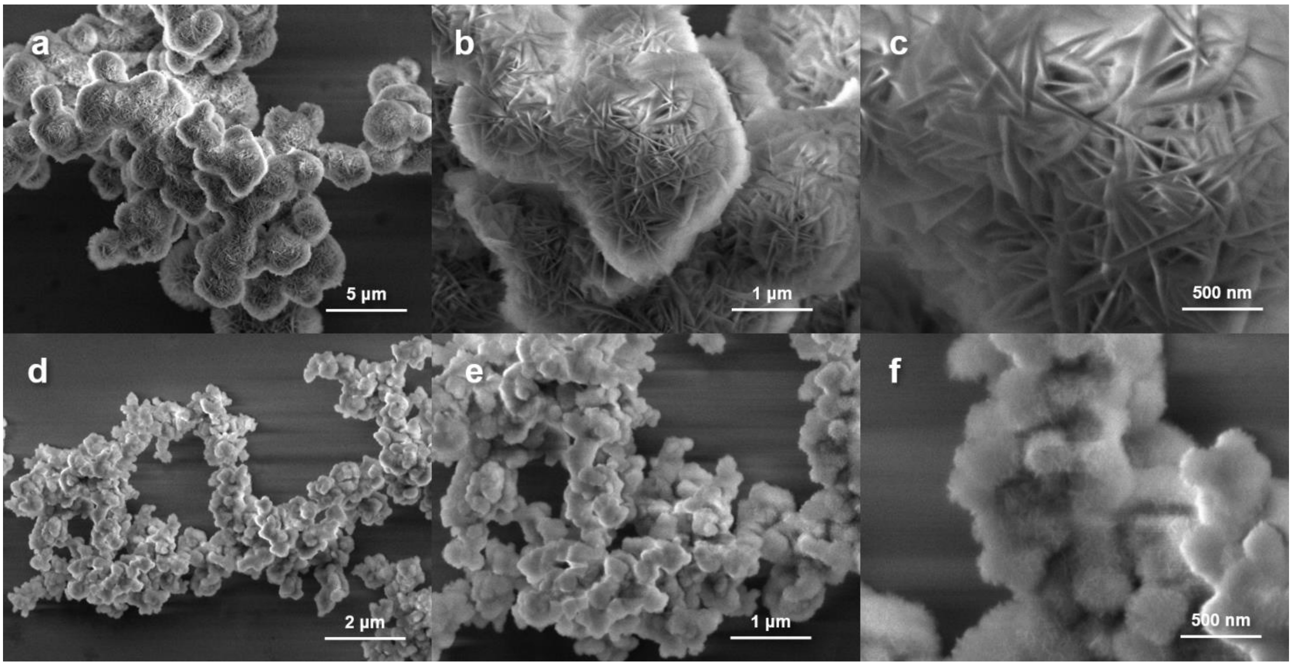

Figure S1. Representative SEM images of (**a-c**) NiFe-LDH and (**d-f**) NiCr-LDH at different scales.

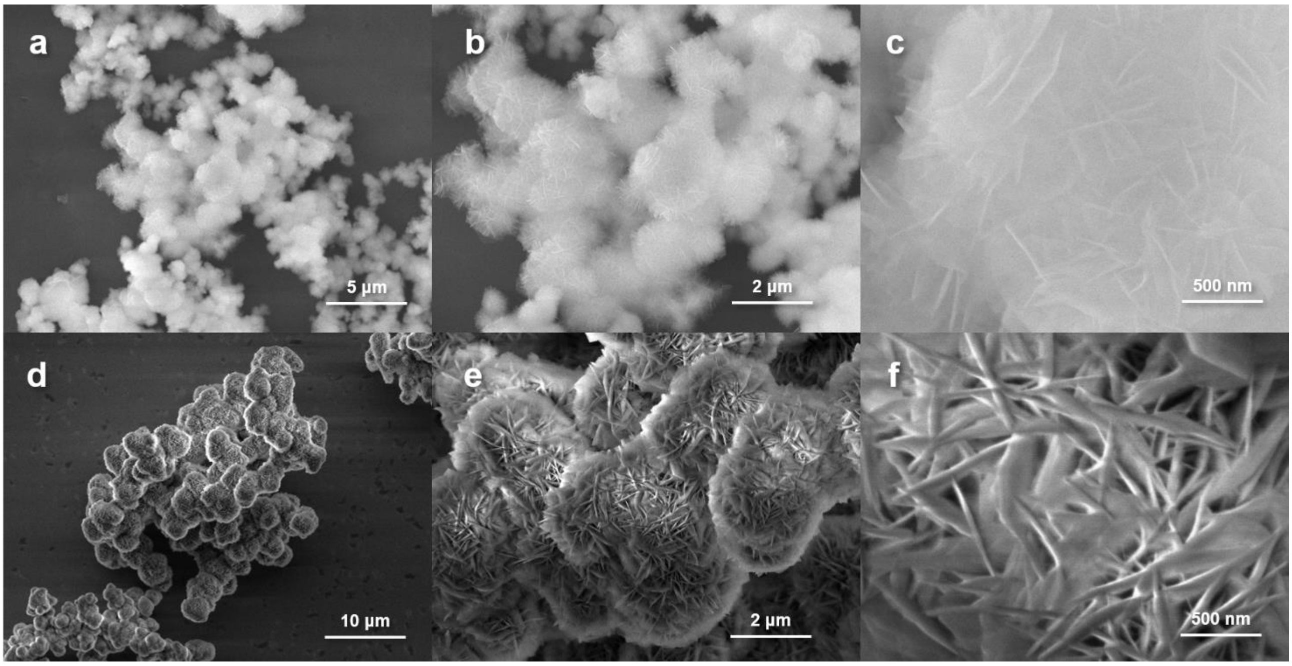

Figure S2. Representative SEM images of (**a-c**) NiCr@NiFe-LDH and (**d-f**) NiCrFe-LTH at different scales.

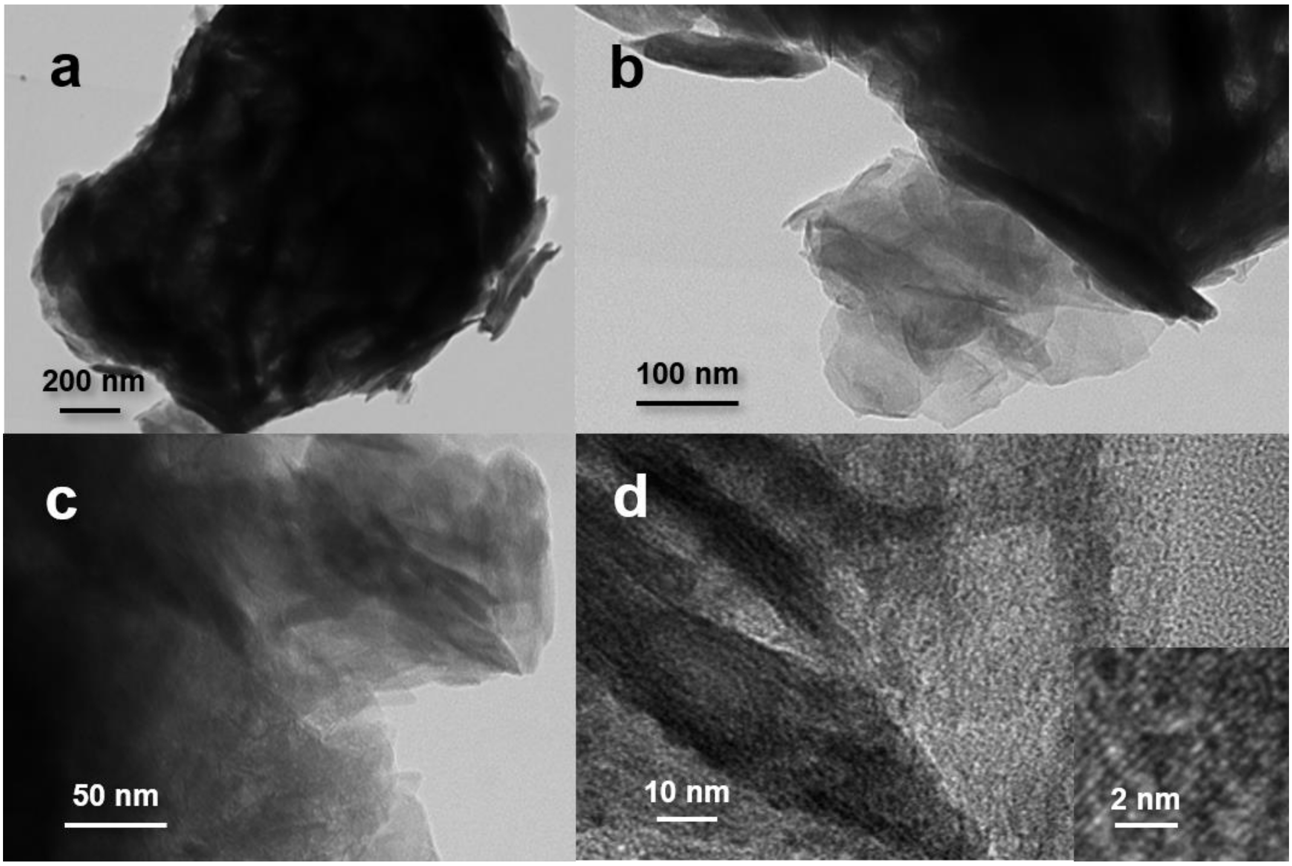

Figure S3. (**a, b**) Representative TEM images of NiCr@NiFe-LDH nanosheets with different magnifications; Representative TEM image (**c**) and (**d**) HR-TEM images with lattice fringes of NiCrFe-LTH nanosheets.

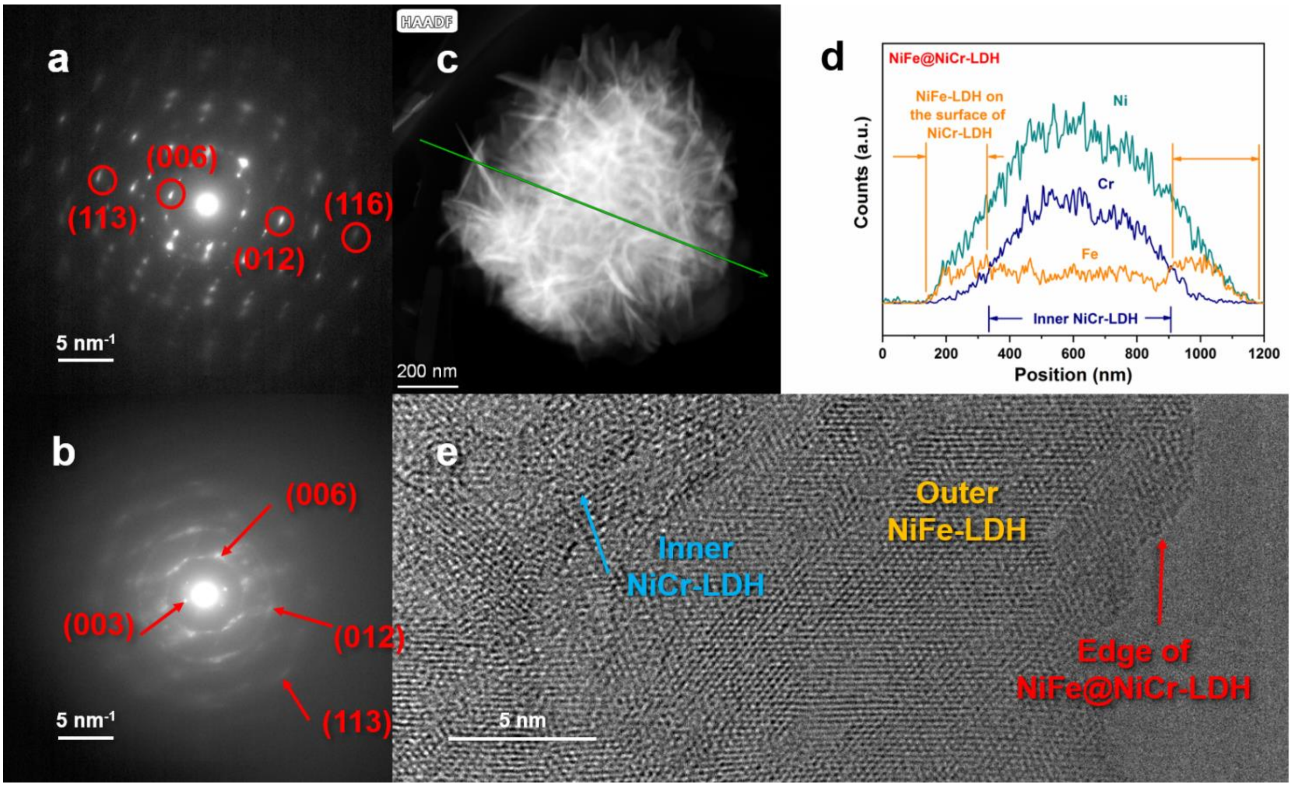

Figure S4. SAED pattern of (**a**) NiCr@NiFe-LDH and (**b**) NiCrFe-LTH; (**c**) The direction marked by a green arrowhead of (**d**) line-scanning profiles of Ni, Cr, and Fe in a NiFe@NiCr-LDH nanosphere; (**e**) HR-TEM images with lattice fringes of NiFe@NiCr-LDH.

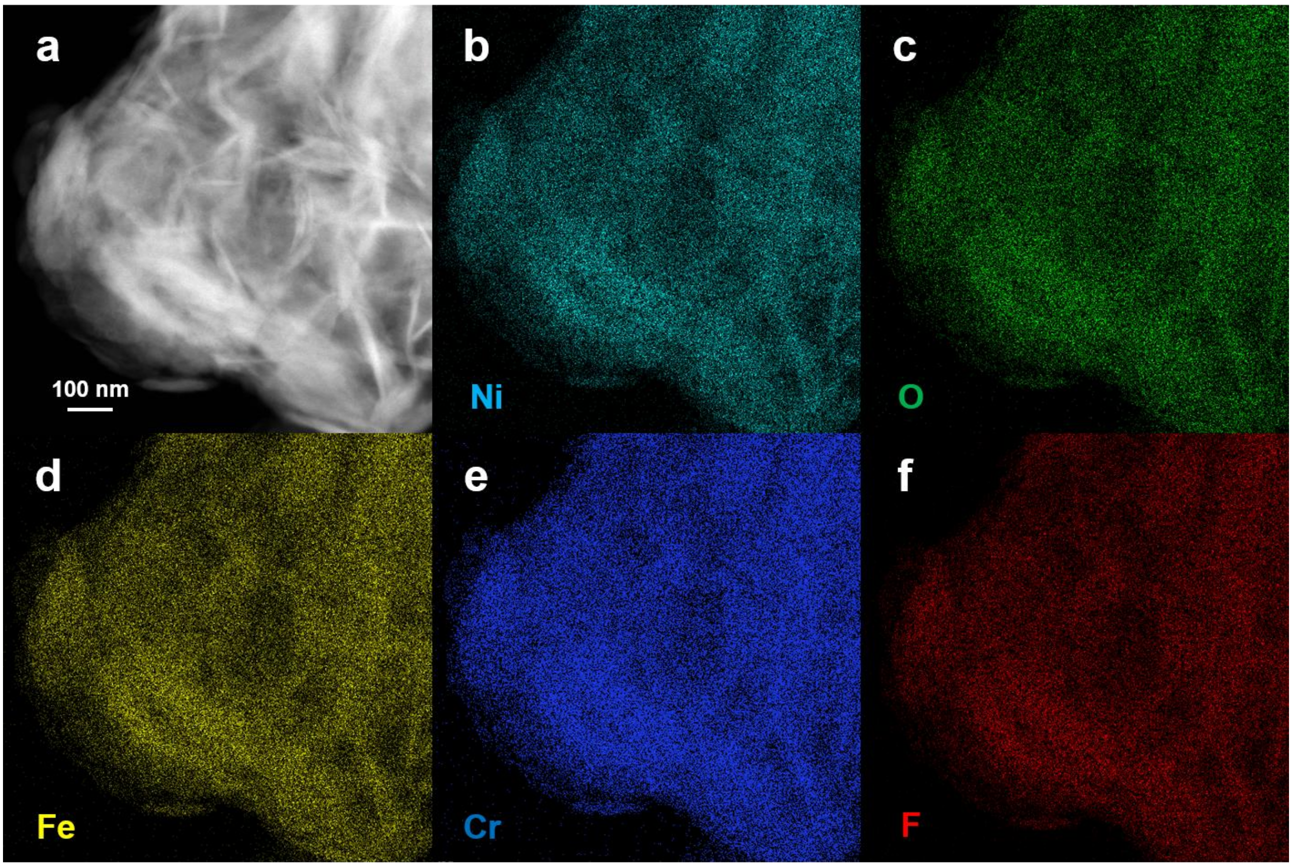

Figure S5. (**a**) High-angle annular dark-field (HAADF) scanning TEM image of a NiCr@NiFe-LDH nanosphere; (**b-f**) Elemental mapping of Ni, O, Fe, Cr, and F of the NiCr@NiFe-LDH nanosphere.

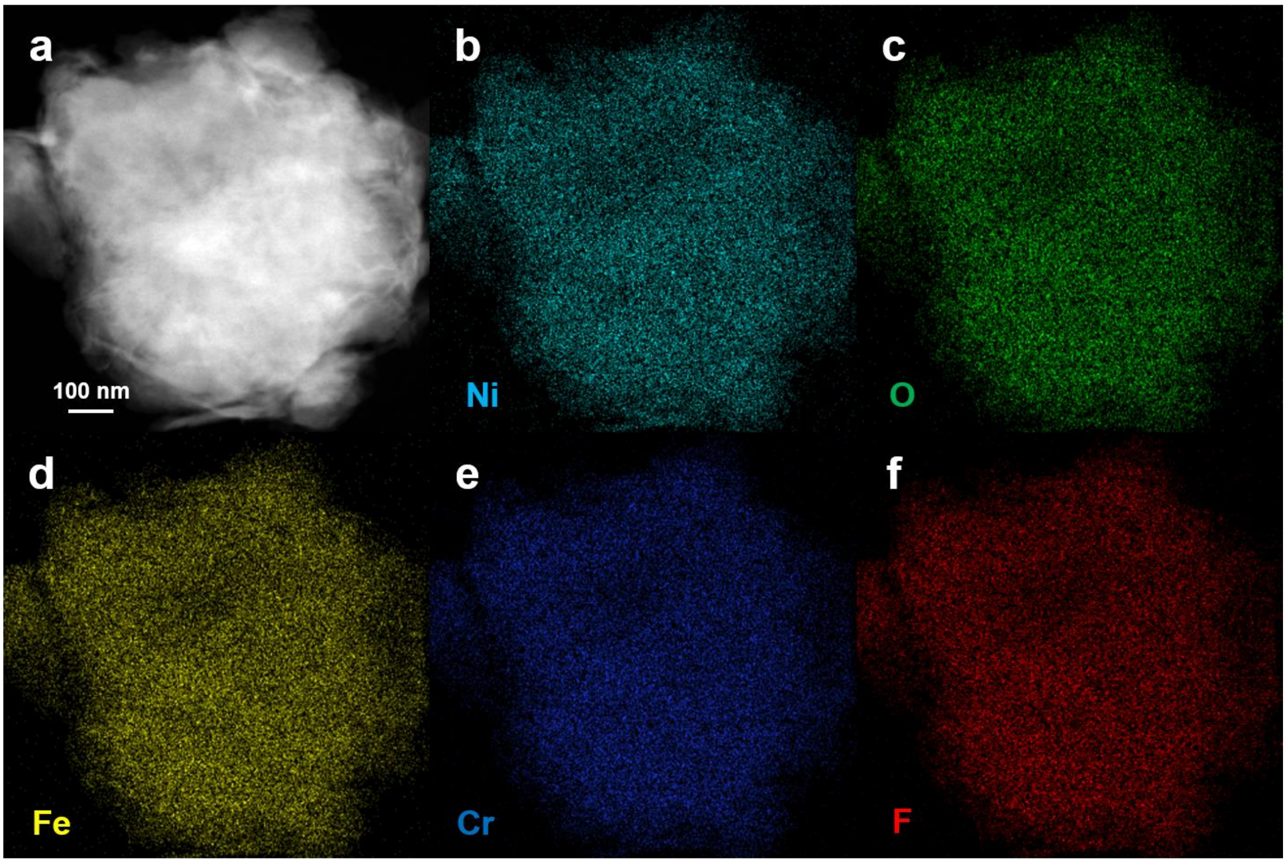

Figure S6. (**a**) High-angle annular dark-field (HAADF) scanning TEM image of a NiCrFe-LTH nanosphere; (**b-f**) Elemental mapping of Ni, O, Fe, Cr, and F of the NiCrFe-LTH nanosphere.

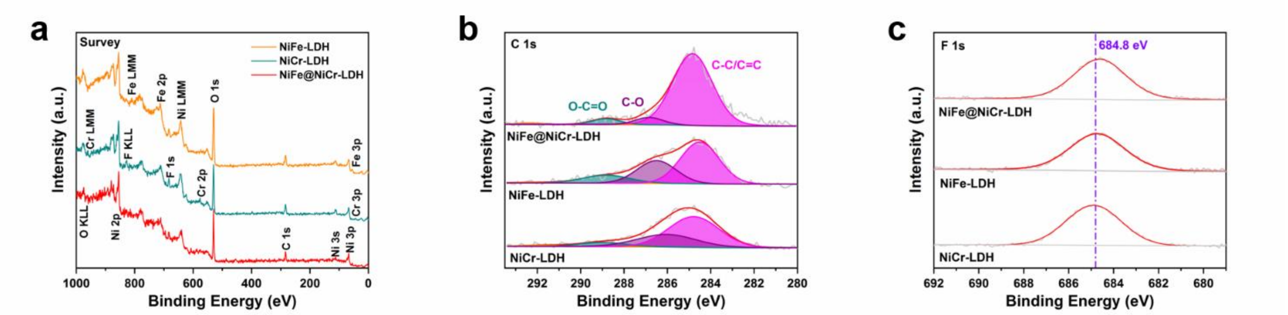

Figure S7. (**a**) Survey scan XPS spectra of NiFe@NiCr-LDH, NiCr-LDH, and NiFe-LDH; High-resolution XPS spectra of the (**b**) C 1s, and (**c**) F 1s electrons in NiFe@NiCr-LDH, NiCr-LDH, and NiFe-LDH.

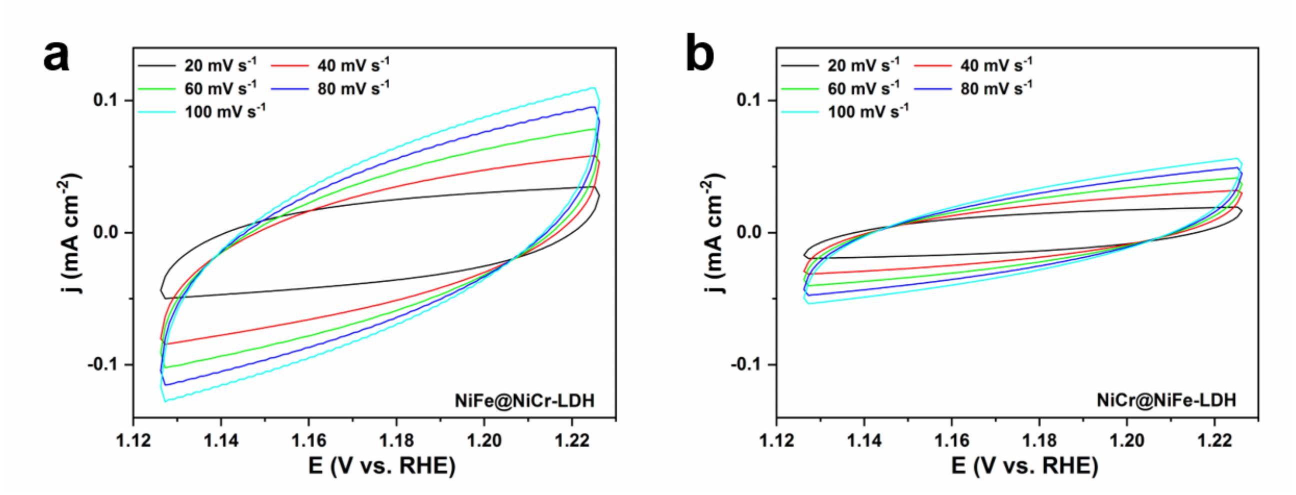

Figure S8. Cyclic voltammograms of (**a**) NiFe@NiCr-LDH and (**b**) NiCr@NiFe-LDH in the region of 1.12-1.22 V in 1.0 M KOH at various scan rates.

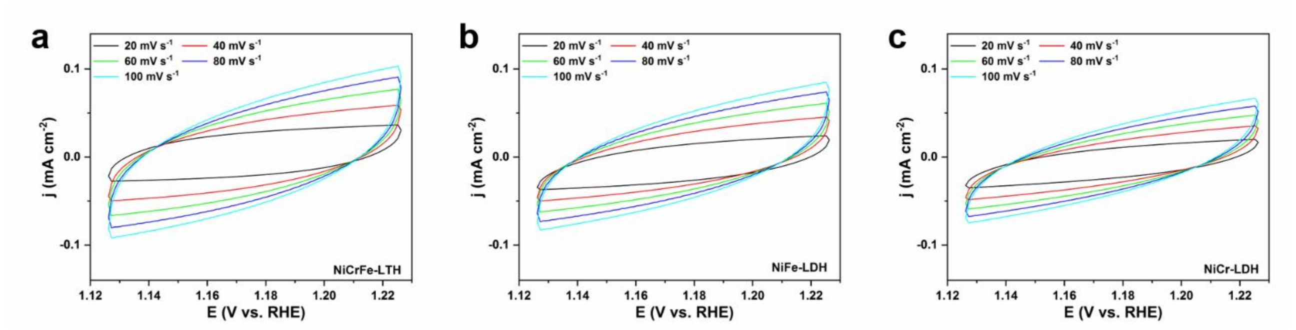

Figure S9. Cyclic voltammograms of (**a**) NiCrFe-LTH, (**b**) NiFe-LDH, and (**c**) NiCr-LDH in the region of 1.12-1.22 V in 1.0 M KOH at various scan rates.

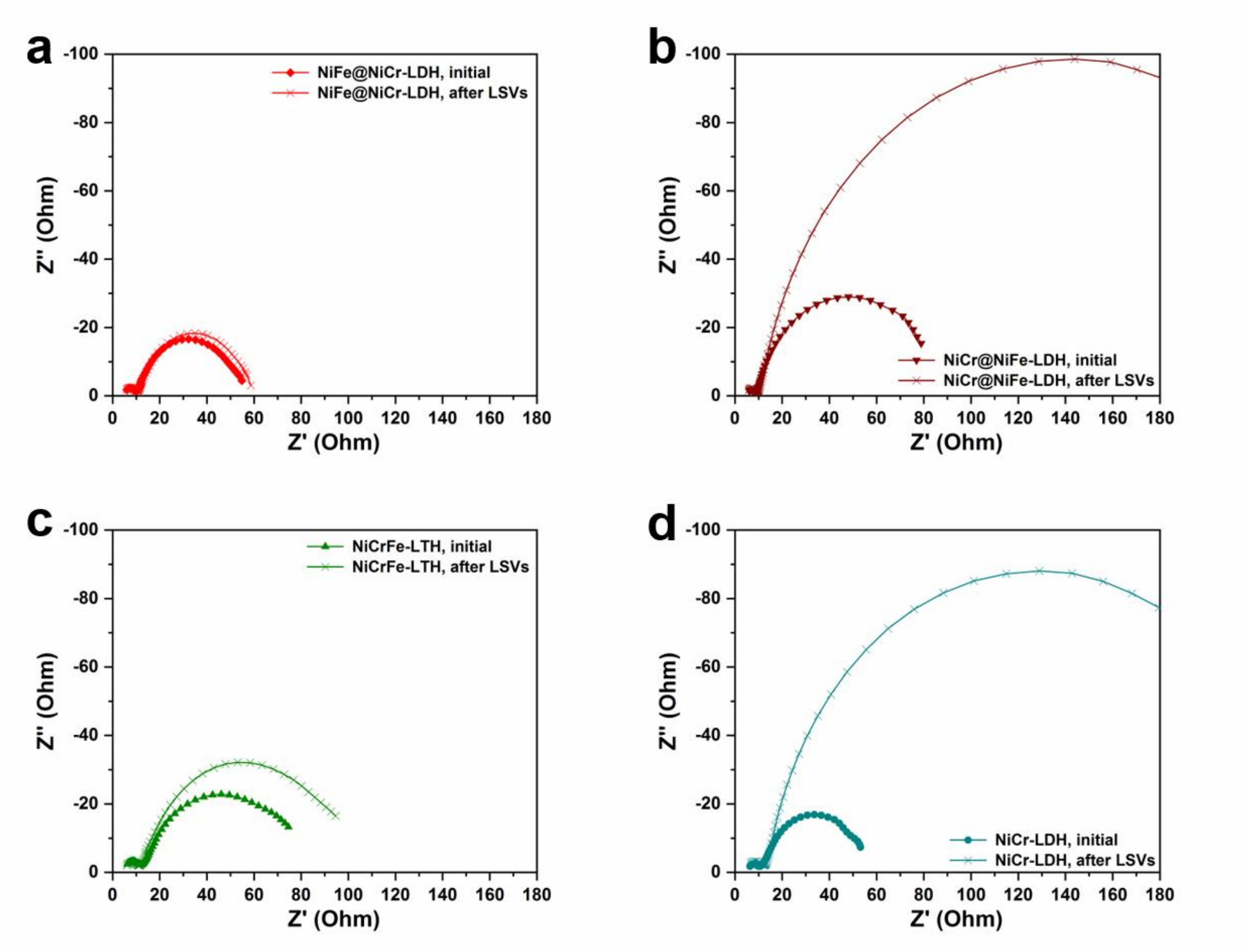

Figure S10 Electrochemical impedance spectra (EIS) of (**a**) NiFe@NiCr-LDH, (**b**) NiCr@NiFe-LDH, (**c**) NiCrFe-LTH, and (**d**) NiCr-LDH before and after the LSV tests.

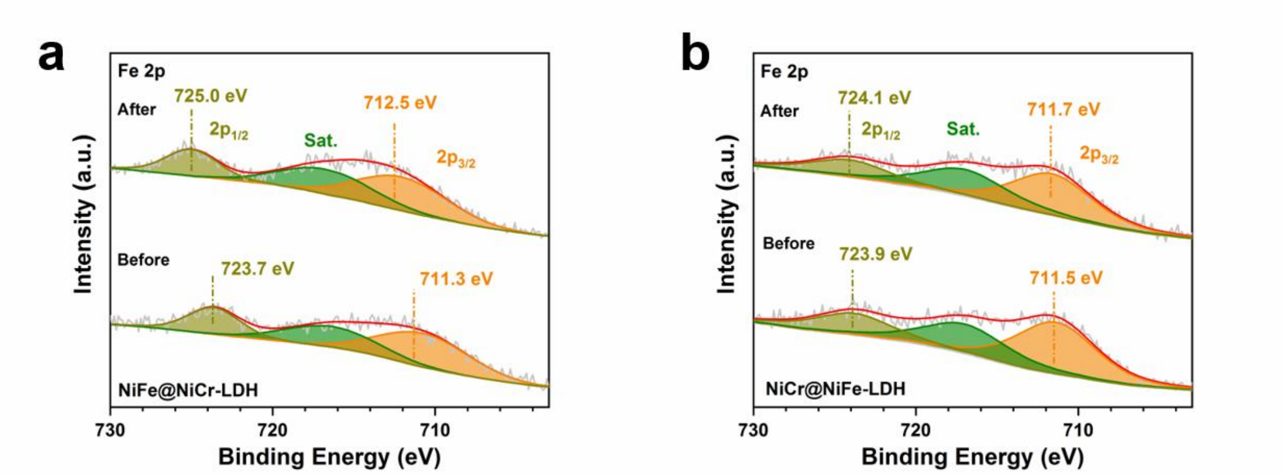

Figure S11 High-resolution XPS spectra of the Fe 2p electrons in (**a**) NiFe@NiCr-LDH and (**b**) NiCr@NiFe-LDH before and after the OER process.

Supplementary Tables

Table S1. The content of Ni, Cr, Fe, and O elements for all the samples, measured by inductively coupled plasma optical emission spectrometry (ICP-OES).

Catalyst	Ni (wt. %)	Cr (wt. %)	Fe (wt. %)	O (wt. %)
NiFe-LDH	40.36	-	13.41	38.03
NiCr-LDH	33.48	14.47	-	43.48
NiCrFe-LTH	35.96	5.10	9.14	39.10
NiCr@NiFe-LDH	38.69	4.22	10.29	38.90
NiFe@NiCr-LDH	35.10	10.99	4.35	41.90

Table S2. The OER activity comparison between NiFe@NiCr-LDH and recently reported LDHs electrocatalysts on glassy carbon electrodes or other substrates in 1.0 M KOH.

LDH catalyst	η_{10} (mV)	Tafel slope (mV dec^{-1})	Reference
NiFe-LDH	306	70	This work
NiCr-LDH	327	104	This work
NiCrFe-LTH	282	68	This work
NiCr@NiFe-LDH	340	99	This work
**NiFe@NiCr-LDH**	**266**	**63**	**This work**
Benchmark RuO_2	249	65	-
NiFeCr-6:2:1	280	131	[2]
NiFeCr-6:2:1-F	283	129	[2]
NiFeCr-LDH/MoS_2	270	85	[3]
CoFeCr-6:2:1	260	41	[4]
3D NiFe-LDH HMS	290	51	[5]
Ni-FeLDH hollow prism	280	49.4	[6]
Co^{2+}-NiFe LDH	265	47	[7]
Cr-FeNi LDH/MXene	232	54.4	[8]

η_{10}: Overpotential at the current density of 10 mA cm^{-2} in the LSV test

Table S3. Summary the fitting parameters of the EIS spectra for OER measured before and after the OER test.

Catalyst	η (mV)	R_s (Ω)	R_{ct} (Ω)	C_{dl} (mF cm^{-2})
NiFe-LDH	236	5.0	159.3	0.39
NiCr-LDH Before OER test	223	4.5	44.1	0.25
**NiCr-LDH After OER test**	**223**	**4.4**	**225.7**	**0.16**
NiCrFe-LTH Before OER test	214	4.9	66.8	0.41
**NiCrFe-LTH After OER test**	**214**	**4.8**	**87.9**	**0.33**
NiCr@NiFe-LDH Before OER test	241	5.0	77.1	0.30
**NiCr@NiFe-LDH After OER test**	**241**	**4.3**	**256.1**	**0.10**
NiFe@NiCr-LDH Before OER test	203	4.8	42.9	0.44
**NiFe@NiCr-LDH After OER test**	**203**	**5.0**	**46.4**	**0.43**

η: Overpotential of the EIS test

R_s: The solution and electrode resistance

R_{ct}: The intrinsic charge transfer resistance of the electrode

$R_{ct\,0}$: The charge transfer resistances at the solid/liquid interface between catalyst and electrolyte

C_{dl}: The capacitance of the double layer of the catalyst

$C_{dl\,0}$: The capacitance of the double layer at the solid/liquid interface between catalyst and electrolyte

References

1. Liu, S.; Wan, R.; Lin, Z.; Liu, Z.; Liu, Y.; Tian, Y.; Qin, D.-D.; Tang, Z., Probing the Co role in promoting the OER and Zn–air battery performance of NiFe-LDH: a combined experimental and theoretical study. *J. Mater. Chem. A* **2022,** 10, (10), 5244-5254.
2. Yang, Y.; Dang, L.; Shearer, M. J.; Sheng, H.; Li, W.; Chen, J.; Xiao, P.; Zhang, Y.; Hamers, R. J.; Jin, S., Highly Active Trimetallic NiFeCr Layered Double Hydroxide Electrocatalysts for Oxygen Evolution Reaction. *Adv. Energy Mater.* **2018,** 8, (15), 1703189.
3. Chen, S.; Yu, C.; Cao, Z.; Huang, X.; Wang, S.; Zhong, H., Trimetallic NiFeCr-LDH/MoS2composites as novel electrocatalyst for OER. *Int. J. Hydrog. Energy* **2021,** 46, (10), 7037-7046.
4. Yang, Y.; Cui, X.; Gao, D.; He, H.; Ou, Y.; Zhou, M.; Lai, Q.; Wei, X.; Xiao, P.; Zhang, Y., Trimetallic CoFeCr hydroxide electrocatalysts synthesized at a low temperature for accelerating water oxidationviatuning the electronic structure of active sites. *Sustain. Energ. Fuels* **2020,** 4, (7), 3647-3653.
5. Zhong, H.; Liu, T.; Zhang, S.; Li, D.; Tang, P.; Alonso-Vante, N.; Feng, Y., Template-free synthesis of three-dimensional NiFe-LDH hollow microsphere with enhanced OER performance in alkaline media. *J. Energy Chem.* **2019,** 33, 130-137.
6. Yu, L.; Yang, J. F.; Guan, B. Y.; Lu, Y.; Lou, X. W. D., Hierarchical Hollow Nanoprisms Based on Ultrathin Ni-Fe Layered Double Hydroxide Nanosheets with Enhanced Electrocatalytic Activity towards Oxygen Evolution. *Angew. Chem. Int. Ed.* **2018,** 130, (1), 178-182.
7. Thenuwara, A. C.; Attanayake, N. H.; Yu, J.; Perdew, J. P.; Elzinga, E. J.; Yan, Q.; Strongin, D. R., Cobalt Intercalated Layered NiFe Double Hydroxides for the Oxygen Evolution Reaction. *J. Phys. Chem. B* **2018,** 122, (2), 847-854.
8. Yan, L.; Du, Z.; Lai, X.; Lan, J.; Liu, X.; Liao, J.; Feng, Y.; Li, H., Synergistically modulating the electronic structure of Cr-doped FeNi LDH nanoarrays by O-vacancy and coupling of MXene for enhanced oxygen evolution reaction. *Int. J. Hydrog. Energy* **2023,** 48, (5), 1892-1903.
